# Supply sensitive services in Swiss ambulatory care: An analysis of basic health insurance records for 2003-2007

**DOI:** 10.1186/1472-6963-10-315

**Published:** 2010-11-23

**Authors:** André Busato, Pius Matter, Beat Künzi, David C Goodman

**Affiliations:** 1 Institute for Evaluative Research in Medicine, University of Bern, Stauffacherstrasse 78, CH-3014, Bern, Switzerland; 2 Department of Economics, University of Bern, Schanzeneckstrasse 1, P.O.B. 8573, CH-3001 Bern, Switzerland; 3 Swisspep - Institute for Quality and Research in Healthcare, Postgasse 17, CH 3011 Bern, Switzerland; 4 The Dartmouth Institute for Health Policy and Clinical Practice, The Center for Health Policy Research, Lebanon, NH 03766, USA

## Abstract

**Background:**

Swiss ambulatory care is characterized by independent, and primarily practice-based, physicians, receiving fee for service reimbursement. This study analyses supply sensitive services using ambulatory care claims data from mandatory health insurance. A first research question was aimed at the hypothesis that physicians with large patient lists decrease their intensity of services and bill less per patient to health insurance, and vice versa: physicians with smaller patient lists compensate for the lack of patients with additional visits and services. A second research question relates to the fact that several cantons are allowing physicians to directly dispense drugs to patients ('self-dispensation') whereas other cantons restrict such direct sales to emergencies only. This second question was based on the assumption that patterns of rescheduling patients for consultations may differ across channels of dispensing prescription drugs and therefore the hypothesis of different consultation costs in this context was investigated.

**Methods:**

Complete claims data paid for by mandatory health insurance of all Swiss physicians in own practices were analyzed for the years 2003-2007. Medical specialties were pooled into six main provider types in ambulatory care: primary care, pediatrics, gynecology & obstetrics, psychiatrists, invasive and non-invasive specialists. For each provider type, regression models at the physician level were used to analyze the relationship between the number of patients treated and the total sum of treatment cost reimbursed by mandatory health insurance.

**Results:**

The results show non-proportional relationships between patient numbers and total sum of treatment cost for all provider types involved implying that treatment costs per patient increase with higher practice size. The related additional costs to the health system are substantial. Regions with self-dispensation had lowest treatment cost for primary care, gynecology, pediatrics and for psychiatrists whereas "prescription only" areas had lowest cost for specialists with non-invasive and invasive activities.

**Conclusions:**

The results indicate that payment methods for services and for prescription drugs are associated with variations in treatment cost that are unlikely warranted by different medical needs of patients alone. Promoting physician accountability of care by linking reimbursements to quality, not quantity, of services are important policy measures to be considered for health care in Switzerland.

## Background

The Swiss health system is based on regulated competition of insurers and providers,[1] and is regarded as one of the role models for American health care reform. Swiss health care shares many characteristics with the United States while also suffering from some similar ills. Ambulatory health care in Switzerland is characterized by independent physicians working mostly in their own practices and are reimbursed on a fee-for-service basis. Patients have direct access to physician specialists with little gate-keeping to regulate access. Hospital ownership is both public and private with payments from cantons and insurers.

Similar to the future mandates of U.S. health care reform, Swiss residents must purchase health insurance individually from competing insurance companies. This mandatory insurance pays for a standardized basic benefit package that includes a wide range of health services[2]. Insurance is offered by almost 100 companies with public subsidizes provided to the less affluent and insurers are not allowed to make profits with basic health insurance. Switzerland's health care expenditures are also near the top of developed countries with 11.6% of GDP. Not surprisingly, during an era of low economic growth, costs and quality are perennial concerns of patients, providers, and policy makers.

This paper examines reimbursements for Swiss ambulatory care. In 2007, ambulatory care services accounted for 22% of total cost in Swiss health care and only 17% of the population had a insurance policy with some form of restricted access to health care providers[3]. Previous research has documented considerable geographic and temporal variation of per capita health care costs in the Swiss ambulatory sector, and several associated factors were identified [4-6]. Other research shows that there might be an optimal list size (i.e. panel size) of patients per general practitioner for providing efficient and high quality care[7]. The present study refines and expands this research by addressing two research questions through the analyses of the complete set of mandatory health insurance consultation claims provided by all ambulatory Swiss physicians during the years 2003-2007 (i.e. of 54% Swiss physician workforce in 2007).

The first question is whether physicians' practice list size is inversely related to the intensity of services and billings per patient; that is, do physicians with shorter patient lists "compensate" with additional services? An association of practice list size and treatment intensity would raise concerns that fee-for-service payment incentivize a higher volume of care without regard to patient needs[8].

The second research question focuses more narrowly on whether prescription drug reimbursement is related to treatment intensity? Several cantons are allowing physicians to directly dispense drugs to patients ('self-dispensation'), while other cantons restrict such direct sales to emergencies only and patients must obtain repeat prescription drugs in a pharmacy ('prescription only'). Some cantons allow both types of dispensation (mixed forms). An association between canton prescription drug dispensing policies and service intensity and consultation costs may be a further indication of a misalignment of reimbursement policies and patient needs.

## Methods

Claims data were obtained from santésuisse, the umbrella organization of Swiss health insurers. Physicians were classified into 49 different medical specialties with board certifications according to the Swiss Medical Association (FMH), together with practice location area codes. Medical specialties were pooled into six main provider types: primary care, pediatrics, gynecology & obstetrics, psychiatrists, invasive (i.e. doing mainly therapeutic operations as e.g. surgery, orthopaedics, nephrology, etc.) and non-invasive specialists (rheumatology, gastroenterology, nephrology, cardiology, etc.) using a classification that was adopted from an earlier project[9]. Consultation records included frequencies and costs categorized by gender of patients, by 20 age groups, and by community of patient residence. Consultation costs were classified by diagnostic and therapeutic activities (treatment cost) and by medication directly dispensed to patients (medication cost) [4]. Data regarding referral to consultants, imaging procedures, hospitals, pharmacies and other services were not available.

The main outcome of this study was the sum of treatment cost reimbursed to physicians by mandatory health insurance. These costs were calculated for each physician by summing up all revenues for diagnostic and therapeutic services (without medication cost) provided during 2003-2007. In addition, the average cost per patient was calculated by dividing the sum of cost by the total number of patients treated during the same period.

This study is exempted from ethic committee review according to Swiss law, no individual patient records were used in the study.

### Data analysis and regression models

Descriptive analyses document the intensity of services in terms of treatment cost per patient across medical provider types, cantons and different drug dispensation practices (Tables 1 and 2).

**Table 1:** Average treatment costs (in Swiss Francs) per patient 2003-2007 across different medical care providers.

Type of provider	**Nr of Cantons**^**a**^	Nr of physicians(% of total)	Average cost perpatient	Interquartile range	**Extremal coefficient**^**b**^
Primary Care	26	8149 (43%)	365.64	67.72	1.80
Gynecology	26	1249 (7%)	259.18	39.70	1.63
Pediatrics	25	972 (5%)	254.56	65.56	1.97
Non-invasive Specialists	26	2716 (14%)	498.19	196.36	4.11
					
Invasive Specialists	26	3039 (16%)	306.92	100.37	2.42
Psychiatrists	25	2986 (16%)	1398.94	417.51	2.84

^a ^Pediatricians and psychiatrists were located in 25 Cantons only

^b ^Ratio of canton with highest vs. canton with lowest cost

**Table 2:** Average treatment cost (in Swiss Francs) per patient 2003-2007 across type of medical provider and different source of drug dispensation.

Type of care	Self dispensation	Mixed forms	Prescription only
Primary Care	345	403 (+14%)^a^	403 (+14%)
Gynecology	256	290 (+12%)	273 (+6%)
Pediatrics	240	266 (+10%)	271 (+11%)
Non-invasive Specialists	635	748 (+15%)	487 (-30%)
Invasive Specialists	326	316 (-3%)	304 (-7%)
Psychiatrists	1368	1726 (+21%)	1897 (+28%)

^a ^difference to self dispensation in brackets

Statistical analyses used the methods described by Grytten and Sorensen[6,10]. We analyzed effects of patient list size on total treatment cost at the physician or practice level using log-log models. In these models, a linear relationship with a unitary slope between patient numbers and treatment costs would indicate no effect of patient list size on costs per patient, whereas slopes below one would indicate that the intensity of services and the related treatment cost per patient decrease with high patient numbers and vice versa. The slope of the regression line (i.e. the location of the supply curve) is thus of central importance as slopes above one may indicate supplier induced demand and slopes below can be seen as an indication of rationing of services.

The main outcomes of these models were the total treatment cost per physician reimbursed by basic health insurance during 2003-2007. The first model was used to estimate the direct relationship between patient number and total treatment cost without adjustment for other cofactors (model 1). Two additional regression models accounting for demographic and geographic factors were used, one model was aimed to assess the overall relationship between the number of patients and total treatments cost (model 2). A third model investigated effects of different patient numbers (quartiles of panel size) on total treatment cost (model 3). Physician data were therefore divided into four groups (quartiles) of patient numbers and each of these groups was studied individually.

### The following relationships describe the four groups

P_Q1 = log(patient number) for ≤ 1st Quartile otherwise P_Q1 = 0

P_Q2 = log(patient number) for > 1st Quartile ≤ median otherwise P_Q2 = 0

P_Q3 = log(patient number) for > median ≤ 3rd Quartile otherwise P_Q3 = 0

P_Q4 = log(patient number) for > 3rd Quartile otherwise P_Q4 = 0

Additional explanatory variables were included in models 2 and 3 in order to analyze effects of patient demographics, geographic location of practices (cantons) and distribution channel of prescription drugs. The structure of the three models is given in table 3.

**Table 3:** Structure of models:

Model 1	Outcome	Total treatment cost per physician for 2003-2007
	Explanatory variable	Total number of patients treated in 2003 - 2007
Model 2	Outcome	Total treatment cost per physician for 2003-2007
	Explanatory variables	Total number of patients treated in 2003 - 2007
		Average age of patients
		Proportion of consultations for female patients^a^
		Type of dispensing drug (prescription only, 'self-dispensation', mixed forms)
		Canton of practice location within type of dispensing prescription drugs (nested effect)

Model 3	Outcome	Total treatment cost per physician for 2003-2007
	Explanatory variables	Quartiles of number of patients treated 2003-2007 (P_Q1 - P_Q4)
		Average age of patients
		Proportion of consultations for female patients^a^
		Type of dispensing drug (prescription only, 'self-dispensation', mixed forms)
		Canton of practice location within type of dispensing prescription drugs (nested effect)

^a ^The variable was omitted in models for gynaecology

Type of dispensing drugs ('prescription only', 'self-dispensation', 'mixed forms') and canton of practice location were treated as classification (i.e. categorical) variables. Cantons were included as nested effects within area of drug dispensation. Results of classified variables were interpreted as least-square means (LS-Means). The Bonferroni procedure was used to adjust for multiple comparisons and exponents of LS-Means were used for tables and graphs. Differences between slopes of quartiles of patient numbers were determined by testing the respective linear combinations of parameters. Residual analyses, performed to validate the statistical procedures, showed no evidence of assumption violations for the models used in the analysis and R-square values exceeded 0.9 for all models specified.

In order to quantify the effect of non-proportional relationships between patient numbers and total practice cost the slope estimate for patient number of model 1 was set to 1 and exponentials of predicted values of this procedure were used to compare cost expected in a setting of proportionality with observed cost. SAS 9.2 (SAS Institute Inc., Cary, NC, USA) and "Proc GLM" were used for all analyses, and the level of significance was set at 0.05 throughout the study.

## Results

### Characteristics of physicians

The distribution of physicians across medical provider types, average treatment costs per patient and data on cantonal variability for 2003-2007 are shown in Table 1. Treatment costs per patient varied markedly across cantons with the magnitude of variation ranging from a 1.6 fold variation for gynecology to a four-fold variation for non-invasive specialists. Average treatment cost per patient showed distinct regional patterns depending on provider type and dispensing channel of prescription drugs (Table 2). Areas with "Self dispensation" had the lowest treatment cost per patient for primary care, gynecology, pediatrics and for psychiatry, whereas "prescription only" areas had lowest cost for specialists with non-invasive and invasive activities.

Notably, in areas with self dispensation, sales of medication accounted for 44% of total primary care practice revenues (sum of reimbursements for treatment and for medication). For specialists, the respective proportions ranged from 11% for psychiatrists to 24% for specialists performing non-invasive treatments. In areas with "prescription only" dispensing the proportion of revenues of directly dispensed medication of total practice revenues ranged from 0.2% in psychiatry to 6% in pediatrics.

### Results of regression models

Unadjusted and adjusted effect estimates of continuous explanatory variables on overall treatment costs at the physicians/practice level are shown in Table 4. Irrespective of number of additional cofactors included in the models, slopes of supply curves (effect of patient numbers on total treatment cost at the practice level) significantly exceeded 1 for all medical provider types, implying that treatment costs per patient increase with higher practice size, i.e. larger practice lists. The effect was particularly strong for psychiatry with a slope estimate of 1.14 in the adjusted model. Furthermore, slope estimates of supply curves of model 1 and model 2 were very similar indicating only minor effects of variables related to patient demographics and practice location (table 4).

**Table 4:** Regression coefficients for total treatment costs at the physicians level 2003 - 2007

	**Model 1**^**a**^		**Model2**^**b**^			
**Type of care**	**Intercept**	**Number of patients**	**Intercept**	**Number of patients**	**Average patient age**	**% consultations for women**

Primary Care	5.370	1.061^c^	3.304	1.064^c^	0.472	-0.044^e^
Gynecology	5.003	1.072^c^	4.228	1.074^c^	0.152	-^d^
Pediatry	5.145	1.046^c^	4.476	1.058^c^	0.131	-0.162
Non-invasive Specialists	5.585	1.057^c^	1.798	1.052^c^	0.815	-0.381
Invasive Specialists	5.233	1.047^c^	3.905	1.040^c^	0.224	-0.125
Psychiatrists	6.582	1.133^c^	5.928	1.143^c^	-0.021^e^	0.022

^a ^Model with number of patients as the only explanatory factor

^b ^Model with number of patients, age, proportion of consultations for female patients, canton and dispensation channel as cofactors

^c ^95% confidence limit is not including 1

^d ^effect was omitted from the model

^e ^non-significant estimates

The overall additional costs associated with these effects are substantial. The difference between the total sums of observed cost for all practices and the respective expected cost under the assumption of proportionality are: Primary care +61%, gynaecology +81%, pediatrics +50%, non-invasive specialists +63%, invasive specialists +49% and psychiatry +116%.

**Figure 1:**
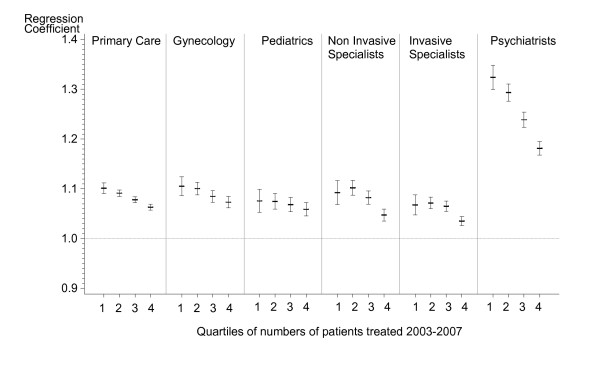
**Regression coefficients^a ^of quartiles of patient panels (model 3)**. aRegression coefficients denote the location of the supply curve and slopes above one indicate inducement of services. Slopes estimates were obtained using linear regression models. Error bars denote 95% confidence intervals of regression coefficients.

Models using quartiles of patient panels indicate that slopes of supply curves generally decrease with higher patient numbers. But slope estimates of quartiles show different patterns across provider types and significant and consistent decreasing slopes for quartiles 1 to 4 i.e. a "dose-response" relationship are seen for primary care and psychiatry only (Figure 1).

**Figure 2:**
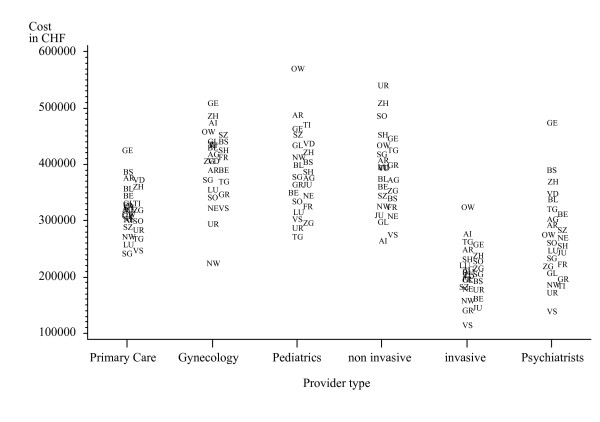
**Cantonal variation of adjusted treatment cost at the practice level across six medical provider typesa**. a Plot symbols denote the abbreviation of canton names and exponents of least square means of regression model 1 are shown as estimates of total treatment costs at the expense of mandatory health insurance.

Adjusted total treatment cost also varied substantially across cantons for all provider types. Cantonal variation of adjusted treatment costs (LS-Means) at the practice level is shown in Figure 2. Significant differences of costs across areas with different channels of drug dispensation were observed for primary care, invasive and non-invasive physicians (Table 5). Pairwise comparisons of least square means across the three dispensation channels indicated significant differences for all possible combinations within these three provider types. However, the extent and direction of differences were not consistent. Adjusted treatment costs in "prescription only" areas were highest for primary care but lowest for both invasive and non invasive care providers.

**Table 5:** Adjusted total sum of treatment costs^a ^in Swiss Francs across medical discipline and group of drug dispensation for 2003-2007.

Type of care	Self dispensation	Mixed forms	Prescription only
Primary Care	296227	321077 (+8%) ^b^	325732 (+10%)
Gynecology^c^	379418	412150 (+9%)	395527 (+4%)
Pediatrics^c^	373214	376611 (+1%)	388110 (+4%)
Non-invasive Specialists	374641	434273 (+16%)	352036 (-6%)
Invasive Specialists	216719	196617 (-9%)	184251 (-15%)
Psychiatrists	235053	274825 (+17%)	268355 (+14)

^a ^Adjusted for cantonal effects, demographic structure and numbers of patients using general, linear models (exponents of LS-means).

^b ^difference to self dispensation in brackets

^c ^no significant differences of LS-Means across groups

## Discussion

Our study demonstrates considerable variation in treatment costs across cantons, by type of drug dispensation, and by the number of patients cared for by each practice. Unexpectedly, larger practice lists were associated with higher per patient costs, in both unadjusted and adjusted models, and for all physician types studied. Furthermore, drug dispensation policies were also associated with differences in treatment costs. These effects are unlikely to be explained by patient needs, and reflect the complex incentives within the Swiss health care system.

### Effects of the health system

The Swiss health system is characterized by both liberalism and federalism with many different stakeholders involved. Cantons have a particularly strong role in regulating and financing health care[2]. The disadvantages of the system are, among others, fragmentation and regional variation in health care expenditures that may not be entirely justified by medical needs of the respective populations [5,11,12]. Our results quantify cantonal variation of treatment cost for physician based ambulatory care, and confirm the impact of cantonal and professional autonomy in providing, coordinating and financing care.

Our results reveal that sales of prescription drugs account for considerable revenues for primary care physicians and, to a lesser degree, for other care providers. Reimbursement data, however, show inconsistent associations with type of drug dispensation across different provider types of care. The dispensation channel of prescription drugs, which is also regulated on a cantonal basis, is an important aspect of the variation in costs. Switzerland is one of the few OECD countries in which doctors are authorized to dispense prescription non-emergency drugs. This policy has been subject of a long debate between physicians, pharmacists and regulators. While self-dispensation may be a financial aid for the struggling primary care physicians, it is may also lead to conflicts in patient and physician interests. Patient perception about benefits of self-dispensation in Switzerland is mixed[13] and it appears that self-dispensation is related to higher prescription volumes in other countries[14].

Variation in service volume that does not reflect patient need has not only direct consequences on health care expenditures, but may trigger a cascade of unnecessary diagnostic tests and other procedures. While there is a sparse literature analyzing potential effects of such events on health outcomes and quality of care [13], there are studies showing that higher treatment intensity does not generally result in better health[15].

### Effects of patient volume on treatment revenues at the practice level

The study confirms and expands earlier research investigating effects of structural attributes of practices on treatment costs and volume in Swiss ambulatory care[5,6]. The overall non-proportional relationships between number of patients and total treatment cost, as shown in Table 4 suggests considerable effects on overall treatment cost of supply induced services. Structural differences such as better practice organization (more staff, better equipment and IT, etc.) that allows for larger treatment volumes may at least partially provide an explanation of such patterns. However, Swiss ambulatory care is based on a fee-for-service system, which may encourage physicians to expand service volumes.

The presence of non-medical incentives to provide higher amounts of care is further supported by decreasing slope estimates of supply curves in practices with large patient populations. These data imply reduction of services at the patient level in these practices or, vice versa, induction of services in practices with fewer patients particularly for psychiatry and to lesser degree for other provider types. Apparently, psychiatric care is sensitive to such mechanisms as it typically lacks the possibilities for expanding services compared to all other provider types, i.e. by simply adding more medical-technical care. The extent to which under treatment or overtreatment occur within these practice settings cannot be ascertained from the present study without further data on health outcomes and patient satisfaction. It is, however, unlikely that the health status of patients in practices with fewer patients differs substantially from practices with more patients. Moreover, our studies control for patient age, one of the dominant factors of morbidity. The findings extend other research [16] that high service production may not translate into improved health of served populations in the ambulatory sector of Swiss health care.

### Limitations and strengths

The observed association between physician activity and treatment costs may be biased by omitted possible confounders, such as individual preferences of patients and physicians. Such associations can only be explored with qualitative research of individual treatment episodes [17]. It is also important to note that our analyses did not fully control for differences in case-mix across the various practices settings. Although age was used as a proxy for health status, case mix may have biased our results particularly in psychiatry where smaller patient numbers can be associated with a higher complexity of cases.

The study does not provide data about the effects of the type drug dispensation scheme on overall expenditures of Swiss ambulatory care. The data that would allow such analyses, including the regional distribution of prescription drug sales of pharmacies and ambulatory hospital departments, are currently not available.

Another limitation is that only data for physician consultations reimbursed by compulsory health insurance were available. Out-of-pocket costs of households accounted for 21% of 2006 overall health care expenditures in Switzerland[3]. It is important, therefore, to note that the results of this study refer only to costs that were paid by mandatory health insurance.

The results may not be compared directly with other countries as health systems differ with respect to structure, financing mechanisms and socio-cultural aspects. The Swiss health care system, however, shares important similarities with the United States - fee-for-service payments, physician owned practices, and large number of insurers. Our findings may help highlight the dilemmas in health care delivery and financing faced by both Switzerland and the United States.

## Conclusions

Our findings reveal that physicians make different decisions as a function of the number of patients they see, and that treatment patterns vary across different systems of providing prescription drugs. The study provides empirical evidence that payment methods for physician services and for prescription drugs are associated with variations in treatment costs that are unlikely to be entirely justified by the medical needs.

## Competing interests

The authors declare that they have no competing interests.

## Authors' contributions

AB obtained the data, performed all statistical analyses and wrote the manuscript, PM provided input on all aspects of health insurance and economics and reviewed the manuscript, BK provided input on ambulatory care and reviewed the manuscript, DCG provided input on research methods and edited the manuscript. All authors read and approved the final manuscript

## Pre-publication history

The pre-publication history for this paper can be accessed here:

http://www.biomedcentral.com/1472-6963/10/315/prepub
